# Prevalence and Distribution of Molar Incisor Hypomineralization (MIH) and Hypomineralized Primary Second Molar (HPSM) Among Children Aged 7-11 Years Residing in Ahmedabad, India: A Prospective, Observational Cross-Sectional Study

**DOI:** 10.7759/cureus.109072

**Published:** 2026-05-18

**Authors:** Shreyash Parmar, Vasudha Sodani, Bhumi Sarvaiya, Vatsal Jani, Manya Vora, Kavya Purohit, Prachi Pandya

**Affiliations:** 1 Pedodontics and Preventive Dentistry, Ahmedabad Dental College and Hospital, Ahmedabad, IND; 2 Pediatric and Preventive Dentistry, Ahmedabad Dental College and Hospital, Ahmedabad, IND; 3 General Dentistry, Muskan Dental Clinic, Ahmedabad, IND; 4 General Dentistry, Shrey Multispeciality Dental Clinic and Implant Center, Patan, IND

**Keywords:** ahmedabad, diagnosis, gujarat, hpsm, hypomineralized primary second molar, india, mih, molar-incisor hypomineralization, pediatric dentistry, prevalence

## Abstract

Background and objective

Molar incisor hypomineralization (MIH) and hypomineralized second primary molars (HPSM) are significant developmental dental anomalies that affect children's quality of life. This study aimed to determine the prevalence, distribution, and correlation between MIH and HPSM among children aged 7-11 years in the five zones of Ahmedabad, Gujarat, India.

Methodology

A cross-sectional screening was conducted involving 1,860 schoolchildren (963 males, 897 females). Ahmedabad was divided into five zones. Inclusion criteria included children aged 7-11 years with parental consent, while children absent on the day of examination or those missing index teeth were excluded. Clinical examinations were performed using the 2021 European Academy of Paediatric Dentistry (EAPD) diagnostic criteria. Following data collection, all categorical data were compiled and statistically analyzed to evaluate the prevalence, clinical severity, and demographic distribution of the anomalies.

Results

The overall MIH prevalence was 2.04% (n = 38) while the dedicated HPSM prevalence was 0.27% (n = 5). Mild severity predominated, accounting for 65.79% of affected cases. The incidence of these developmental anomalies was entirely gender-neutral (*p *= 0.080 for MIH; *p *= 0.6769 for HPSM) and independent of specific age groups within the 7-11-year range (*p *= 0.984).

Conclusions

MIH and HPSM demonstrated a low but clinically significant prevalence within this cohort, with no statistically significant associations observed with gender or age groups. Despite the relatively low prevalence, the profound clinical consequences of these enamel defects highlight the critical need for early identification, heightened clinical vigilance, and timely preventive care.

## Introduction

Molar incisor hypomineralization (MIH) is defined as a systemic-origin, qualitative enamel defect affecting one to four first permanent molars and frequently involving the permanent incisors [1]. A similar developmental defect in the primary dentition is termed hypomineralized second primary molars (HPSM). Clinically, these anomalies present as demarcated opacities that range in color from white to yellow or brown. Because the hypomineralized enamel is porous and fragile, affected teeth are highly susceptible to post-eruptive enamel breakdown and severe dentin hypersensitivity. This structural compromise complicates oral hygiene practices and significantly elevates the risk of early, rapid dental caries progression [1, 2]. The global clinical burden of this condition is substantial, although the precise etiology remains complex and multifactorial, and is frequently linked to early childhood systemic illnesses, environmental disruptions, or medical complications rather than fixed genetics [3].

Globally, the prevalence of MIH varies widely, with recent systematic reviews estimating a global burden of between 13.1% and 15.5% [4,5]. Regionally, the prevalence across the Asian continent is reported at around 13.7% [4]. In India, a heavily populated and demographically diverse nation, recent meta-analyses estimate a pooled MIH prevalence of approximately 10% - a figure supported by various regional studies across the country [6,7].

Within the state of Gujarat, epidemiological data show wide regional variation that warrants further investigation. Recent data from the city of Bhavnagar indicated a comparatively lower prevalence of 2.45%, while a study in Gandhinagar found a significantly higher prevalence of 9.2% [8,9]. Despite these contrasting reports, the exact localized disease burden and prevalence data for children aged 7-11 years living in Ahmedabad, the largest urban center in the state, remain completely unreported. Establishing baseline epidemiological data in such a major demographic hub is critical for the allocation of appropriate pediatric dental resources. Therefore, this study aimed to (1) determine the prevalence of MIH and HPSM, (2) assess their distribution according to age, gender, and geographic zones, and (3) evaluate the association between MIH and HPSM among children aged 7-11 years in Ahmedabad.

## Materials and methods

Study design and setting

A prospective observational cross-sectional study was conducted from January 1, 2026, to February 7, 2026, to determine the prevalence and distribution of MIH and HPSM among schoolchildren. Ahmedabad was divided into five administrative zones. A comprehensive list of primary schools was obtained for each zone, and schools were selected using a stratified random sampling method to ensure proportional geographic representation. Within the selected schools, children aged 7-11 years were randomly sampled from class rosters. To account for potential clustering effects at the school level, the sample allocation was kept approximately equal across all five zones, ensuring a well-balanced and representative cohort. Clinical screenings were performed on the school premises in natural daylight using disposable tongue depressors.

Ethical considerations

The study protocol was reviewed and approved by the Institutional Ethics Committee of Ahmedabad Dental College (IECADC) (approval number IECADC/153/2025). Before the commencement of the study, official consent and permission were obtained from the respective school authorities. The complete screening procedure was thoroughly explained to all participating children to obtain their verbal assent, in addition to the formal informed consent provided by the school authorities.

Study population and sample size

The study population comprised children aged 711 years residing in Ahmedabad. The required sample size was determined prior to the study using G*Power software (Version 3.1) with the parameters set at an effect size (w) of 0.1, an alpha error probability of 0.05, and a statistical power (1-β) of 0.95. This calculation yielded a minimum target sample size of 1,858 subjects. To ensure adequate regional representation, the study utilized a stratified random sampling methodology, recruiting approximately 372 children from each of the five administrative zones. A total of 1,860 children, comprising 963 males and 897 females, were successfully recruited and examined.

Inclusion and exclusion criteria

Children aged 7-11 years who were cooperative for the dental examination and had approval from the school authorities were included. Children who were absent on the day of examination, those missing the required index teeth, or those undergoing active orthodontic treatment with fixed appliances that obscured enamel surfaces were excluded.

Clinical examination and diagnostic criteria

All clinical examinations were performed exclusively by a single trained investigator to eliminate inter-examiner variability and ensure diagnostic consistency. Standard infection control measures were strictly maintained throughout the study. During screening, the 12 index teeth (permanent first molars and incisors), along with the primary second molars, were carefully evaluated. Children were examined while seated on standard school chairs positioned to avoid direct sunlight, optimizing visualization under natural daylight. Clinical visual examinations were performed using tongue depressors.

The teeth were examined in their natural, wet state; no prior tooth preparation, drying with compressed air, or professional dental prophylaxis was performed, reflecting standard field epidemiological conditions. The diagnosis of enamel defects was conducted in strict adherence to the updated 2021 European Academy of Paediatric Dentistry (EAPD) best clinical practice guidelines [10]. The diagnostic thresholds were operationalized as follows: 'mild' (demarcated opacities without enamel breakdown), 'moderate' (enamel breakdown limited to one or two surfaces without cuspal involvement), and 'severe' (widespread post-eruptive breakdown, cuspal involvement, extraction due to MIH or atypical restorations).

Examiner calibration and reliability

Before the commencement of the study, the single clinical examiner underwent a standard calibration process. This involved evaluating a set of clinical photographs displaying various enamel defects, including MIH and HPSM, according to EAPD criteria. The examiner re-evaluated the same photographs after a two-week interval to assess consistency. Intra-examiner reliability was measured using Cohen’s Kappa statistic, yielding a score of 0.89, indicating excellent diagnostic consistency. This high reliability supports the single-examiner approach, which was utilized to eliminate inter-examiner variability.

Statistical analysis

Children who were absent on the day of examination, lacked parental consent, or had missing index teeth were excluded from the final dataset. Following data collection, all categorical data were initially compiled into a Microsoft Excel spreadsheet, where they were validated and cross-checked for missing variables. Statistical analyses were performed using IBM SPSS Statistics for Windows, Version 26 (Released 2019; IBM Corp., Armonk, NY). Chi-square and Fisher's exact tests were utilized to assess differences between categorical variables, with the level of statistical significance set at *p* < 0.05.

## Results

A cross-sectional screening of 1,860 schoolchildren (963 males and 897 females) aged 7-11 years was conducted across the five zones of Ahmedabad. Developmental anomalies were observed, with an overall MIH prevalence of 2.04% and HPSM prevalence of 0.27% (Table 1).

**Table 1:** Prevalence of MIH and HPSM MIH: molar incisor hypomineralization; HPSM: hypomineralized primary second molar

Anomalies	Present, n (%)	Absent, n (%)	Prevalence
MIH	38 (2.04)	1822 (97.96)	2.04%
HPSM	5 (0.27)	1855 (99.73)	0.27%

**Figure 1:**
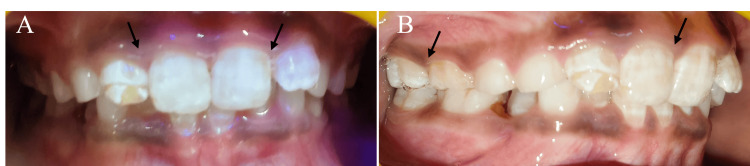
Clinical presentation of mild MIH (A) Anterior view demonstrating demarcated creamy-white opacities on the permanent incisors, and (B) lateral view in occlusion, highlighting affected teeth MIH: molar incisor hypomineralization

**Figure 2:**
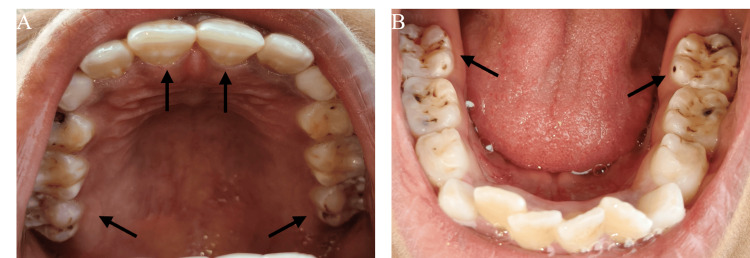
Clinical presentation of moderate MIH (A) Maxillary arch and (B) mandibular arch. Arrows indicate the affected first permanent molars exhibiting moderate demarcated opacities with localized structural enamel involvement MIH: molar incisor hypomineralization

**Figure 3:**
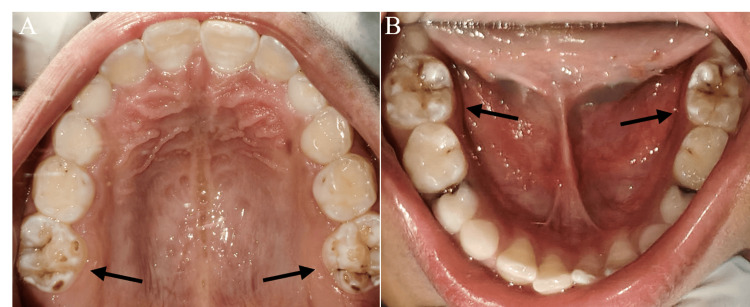
Clinical presentation of severe MIH (A) Maxillary arch exhibiting severe MIH and (B) mandibular arch demonstrating moderate to severe MIH. Arrows indicate the affected teeth MIH: molar incisor hypomineralization

Regarding clinical severity, mild cases predominated significantly, accounting for 65.79% of the diagnosed MIH cases. Moderate severity was observed in 28.95%, and severe MIH was recorded in 5.26% (Table 2). The clinical presentations of mild, moderate, and severe MIH are illustrated in Figures 1-3.

**Table 2:** Clinical severity of MIH cases MIH: molar incisor hypomineralization

Anomaly	Severity, n (%)		
	Mild	Moderate	Severe
MIH	25 (65.79)	11 (28.95)	2 (5.26)

The demographic distribution of these defects revealed no statistically significant gender predilection (Tables 3, 4); MIH was present in 25 males (2.60%) and 13 females (1.45%). Statistical analysis confirmed that the occurrence of both MIH (*p* = 0.080) and HPSM (*p* = 0.6769) was entirely gender-neutral and showed no statistically significant (*p* < 0.05) differences between males and females.

**Table 3:** Gender-wise distribution and statistical association of MIH *p*-values were calculated using the Chi-square test. The Chi-square statistic (χ²) is reported. Statistical significance was set at *p* < 0.05 MIH: molar incisor hypomineralization

Anomaly	Gender	Present, n (%)	Absent, n (%)	Chi-square (χ2)	*p*-Value
MIH	Male	25 (2.60)	938 (97.40)	3.055	0.080
	Female	13 (1.45)	884 (98.55)		

**Table 4:** Gender-wise distribution and statistical association of HPSM *p*-values were calculated using Fisher's exact test. The odds ratio is reported. Statistical significance was set at *p* < 0.05 HPSM: hypomineralized primary second molar

Anomaly	Gender	Present, n (%)	Absent, n (%)	Odds ratio	*p*-Value
HPSM	Male	2 (0.21)	961 (99.79)	0.6202	0.6769
	Female	3 (0.33)	894 (99.67)		

The trend across age cohorts was stable, with group-specific prevalence rates fluctuating mildly between 1.69% in seven-year-olds and an apex of 2.33% in nine-year-olds (Table 5). The absolute volume of cases peaked in the nine-year-old cohort (10 cases). The uniform prevalence observed across all age groups statistically confirms that MIH is a completely age-independent, pre-eruptive systemic event rather than an actively spreading disease.

**Table 5:** Age-wise distribution and statistical association of MIH *p*-values were calculated using the Chi-square test. The Chi-square statistic (χ²) is reported. Statistical significance was set at *p* < 0.05 MIH: molar incisor hypomineralization

MIH	N	7 years, n (%)	8 years, n (%)	9 years, n (%)	10 years, n (%)	11 years, n (%)	Chi-square (χ2)	*p*-Value
Present	38	4 (1.69)	7 (1.97)	10 (2.33)	8 (1.92)	9 (2.14)	0.373	0.984
Absent	1822	233 (98.31)	349 (98.03)	420 (97.67)	408 (98.08)	412 (97.86)		

## Discussion

In pediatric dentistry, MIH and HPSM pose significant clinical challenges. A strong association exists between these developmental defects and rapid post-eruptive enamel breakdown, severe hypersensitivity, and an increased risk of dental caries [1,8]. In the present study, the overall prevalence of MIH among children aged 7-11 years in Ahmedabad was 2.04%, alongside an HPSM prevalence of 0.27%. This observed MIH prevalence of 2.04% is notably lower than the estimated global burden. Comprehensive meta-analyses indicate that the worldwide pooled prevalence of MIH ranges from 12.9% to 15.5% [2,3,11]. In contrast to these higher international figures, the disease burden in Ahmedabad is substantially lower than the prevalence reported in cross-sectional studies from Italy, Western Australia, Sudan, Austria, and Brazil [12-16]. A correspondingly low prevalence of 2.8% has been reported among children in Hong Kong, suggesting concordance with the results of the present study [17].

When contextualized within the national landscape, the results of the study are significantly lower than the pooled MIH prevalence of 10.0% estimated for the overall Indian pediatric population by Shetty et al. [4]. Epidemiological data demonstrate a wide variance across different regions of India. The 2.04% prevalence in Ahmedabad is markedly lower than the rates reported in several northern, western, and southern Indian cities such as Ludhiana (7.20%), Udaipur (9.46%), Solan (2.9%), Chandigarh (6.31%), and Chennai (9.7%) [5,18-21]. Conversely, our findings are slightly higher than the remarkably low disease burdens recorded in Central Delhi (1.17%) and Bengaluru (0.48%) [22,23].

Focusing specifically on data within the state of Gujarat, our 2.04% MIH prevalence closely parallels the 2.45% prevalence documented in the neighboring city of Bhavnagar [6]. In stark contrast, earlier reports from the city of Gandhinagar indicated a significantly higher regional prevalence of 9.2% [7]. These pronounced regional disparities within the same state suggest that variations in the reported disease burden may be influenced by highly localized environmental exposures, distinct systemic etiological factors during early enamel maturation, or specific pediatric cohort characteristics [9].

Regarding clinical severity, the predominance of mild MIH cases (65.79%) in Ahmedabad is highly consistent with epidemiological trends reported in Bhavnagar. This indicates that the vast majority of affected teeth in this geographic region present with mild demarcated opacities rather than severe structural breakdown [6]. This pattern of predominantly mild defects is strongly supported by national findings from Ludhiana and Chandigarh (61.9% and 85%, respectively) as well as international cohorts in Austria and Brazil (71% and 66.2%-73.1%, respectively), where mild opacities without post-eruptive breakdown were the most frequently observed clinical presentation [5,15,16,20]. Demographically, the study found no statistically significant association between the occurrence of MIH or HPSM and gender. While this complete gender neutrality contradicts the distinct female predilection observed in Bhavnagar, Lopes et al. and Zhao et al. have concluded that there is no statistically significant difference in prevalence between males and females [6,11,24].

Other Indian studies evaluating populations in Ludhiana, Central Delhi, and Chandigarh similarly reported no significant association between gender and MIH prevalence [5,20,23]. Similarly, no significant variations were observed across the assessed age groups. While this indicates a uniform distribution across the cohort, the statistical power of these specific subset analyses is limited by the relatively small absolute number of positive cases. This uniform presentation generally aligns with the concept of MIH as a pre-eruptive developmental anomaly, even though cross-sectional epidemiological data cannot definitively confirm the specific underlying pathophysiological pathways [1,9].

The early identification of HPSM serves as a crucial predictive marker for impending MIH in the permanent dentition. Because a strong positive association between MIH and an increased dental caries experience has been firmly established, there is an absolute necessity for early identification and intervention [8]. A timely and proactive diagnostic approach utilizing the updated EAPD best clinical practice guidelines is highly recommended [10]. By implementing appropriate evidence-based treatment modalities ranging from resin pits and fissure sealants to glass ionomer cements for mild cases to full coverage restorations for severe breakdowns, practitioners can restore normal masticatory function, effectively manage hypersensitivity, and maximize tooth longevity [25].

When compared with the broader literature, the observed MIH prevalence of 2.04% and HPSM prevalence of 0.27% in the present study were notably lower than global averages. Large-scale reviews by Schwendicke et al. and Zhao et al. reported a worldwide MIH prevalence ranging from 11% to 14.2% [3,11]. The findings of the present study closely align with other regional Indian studies. Subramaniam et al. reported a similarly low prevalence of 0.48% among children in Bengaluru [22]. This discrepancy between global averages and regional Indian data suggests that geographic, environmental, and local etiological factors may substantially influence the occurrence of MIH.

Regarding clinical severity, the predominance of mild MIH cases (65.79%) in the present study was highly consistent with the findings of Mittal et al., who also reported demarcated white or creamy opacities without post-eruptive enamel breakdown as the most common clinical presentation among Indian schoolchildren [20]. The absence of a statistically significant gender predilection (*p* = 0.080) observed in the present study is consistent with the findings of several international studies and systematic reviews that reported no significant difference in MIH prevalence between males and females [11,24].

Limitations of the study

The cross-sectional design of the present study limited the ability to establish causal relationships between potential etiological factors and the occurrence of MIH and HPSM. Although standardized diagnostic criteria were followed, the examinations were conducted under field conditions in school settings using natural daylight, which may have been less sensitive than examinations performed in a fully equipped clinical environment. Teeth were examined in a wet condition without professional prophylaxis or air drying, which may have resulted in underdiagnosis of mild demarcated opacities. The relatively low number of positive cases also reduced the statistical power for subgroup analyses. Variables such as detailed prenatal and postnatal medical history, environmental exposures, and socioeconomic status were not assessed in the present study. Although the stratified sampling method provided representative coverage across Ahmedabad, the findings may not be generalizable to broader populations or different geographic regions.

Future scope

Future multicentric, longitudinal studies involving larger and more diverse populations are recommended to better understand the epidemiology and etiological factors associated with MIH and HPSM. Incorporating detailed medical histories, environmental risk assessments, and socioeconomic variables may help identify potential contributing factors and improve preventive strategies. Additionally, studies utilizing standardized photographic documentation and controlled clinical examination conditions may enhance diagnostic accuracy and facilitate more reliable comparisons between regional and international data.

## Conclusions

This cross-sectional study establishes important baseline epidemiological data for Ahmedabad, revealing a low but clinically significant prevalence of MIH (2.04%) and HPSM (0.27%) among children aged 7-11 years. The localized disease burden predominantly consisted of mild demarcated opacities with no statistically significant associations observed regarding gender or age groups. Despite the relatively low prevalence, the profound clinical consequences of these defects highlight the critical need for early identification, heightened clinical vigilance, and timely preventive care.
